# Convolutional Neural Network Based on Fluorescein Angiography Images for Retinopathy of Prematurity Management

**DOI:** 10.1167/tvst.9.2.37

**Published:** 2020-07-07

**Authors:** Domenico Lepore, Marco H. Ji, Monica M. Pagliara, Jacopo Lenkowicz, Nikola D. Capocchiano, Luca Tagliaferri, Luca Boldrini, Vincenzo Valentini, Andrea Damiani, for the FA Team of the Italian ROP Study Group

**Affiliations:** 1 Dipartimento di Oftalmologia, Fondazione Policlinico Universitario “A. Gemelli” IRCCS, Rome, Italy; 2 Byers Eye Institute, Horngren Family Vitreoretinal Center, Department of Ophthalmology, Stanford University School of Medicine, Palo Alto, CA, USA; 3 UOC Radioterapia Oncologica, Dipartimento di Diagnostica per Immagini, Radioterapia Oncologica ed Ematologia, Fondazione Policlinico Universitario “A. Gemelli” IRCCS, Rome, Italy

**Keywords:** retinopathy of prematurity, deep leaning, fluorescein angiography

## Abstract

**Purpose:**

The purpose of this study was to explore the use of fluorescein angiography (FA) images in a convolutional neural network (CNN) in the management of retinopathy of prematurity (ROP).

**Methods:**

The dataset involved a total of 835 FA images of 149 eyes (90 patients), where each eye was associated with a binary outcome (57 “untreated” eyes and 92 “treated”; 308 “untreated” images, 527 “treated”). The resolution of the images was 1600 and 1200 px in 20% of cases, whereas the remaining 80% had a resolution of 640 and 480 px. All the images were resized to 640 and 480 px before training and no other preprocessing was applied. A CNN with four convolutional layers was trained on 90% of the images (*n* = 752) randomly chosen. The accuracy of the prediction was assessed on the remaining 10% of images (*n* = 83). Keras version 2.2.0 for R with Tensorflow backend version 1.11.0 was used for the analysis.

**Results:**

The validation accuracy after 100 epochs was 0.88, whereas training accuracy was 0.97. The receiver operating characteristic (ROC) presented an area under the curve (AUC) of 0.91.

**Conclusions:**

Our study showed, we believe for the first time, the applicability of artificial intelligence (CNN) technology in the ROP management driven by FA. Further studies are needed to exploit different fields of applications of this technology.

**Translational Relevance:**

This algorithm is the basis for a system that could be applied to both ROP as well as experimental oxygen induced retinopathy.

## Introduction

Inter-expert and even intra-expert agreement are two of the major problems in the management of all clinical activities related to retinopathy of prematurity (ROP). Chiang et al. and Wallace et al. in 2007 and 2008 showed high level of inconsistency even among experts in ROP diagnosis.^1^^,^^2^ Variability in the features considered during the cognitive process for diagnosis is one of the major causes of disagreement. Bolón-Canedo et al. in 2015 demonstrated that an automated diagnosis system based on the machine learning technique could improve diagnostic accuracy for ROP and especially standardization among clinicians.^3^ More recently, Brown et al. developed and trained a computer-based image analysis to classify 5511 fundus images of prematurely born babies (preemies) and compared them to reference standard diagnosis (RSD), achieving a diagnostic performance similar or better than human ROP experts.^4^ All aforementioned systems were based on regular color fundus images.

The aim of this study was to demonstrate the applicability of fluorescein angiography (FA) for the first time in a convolutional neural network (CNN) for the management of ROP.

## Methods

This study was approved by the institutional review boards at all participating centers and was conducted in accordance with the tenets of the Declaration of Helsinki. Informed consent was obtained from the parents or guardians for all enrolled patients.

**Figure 1.**
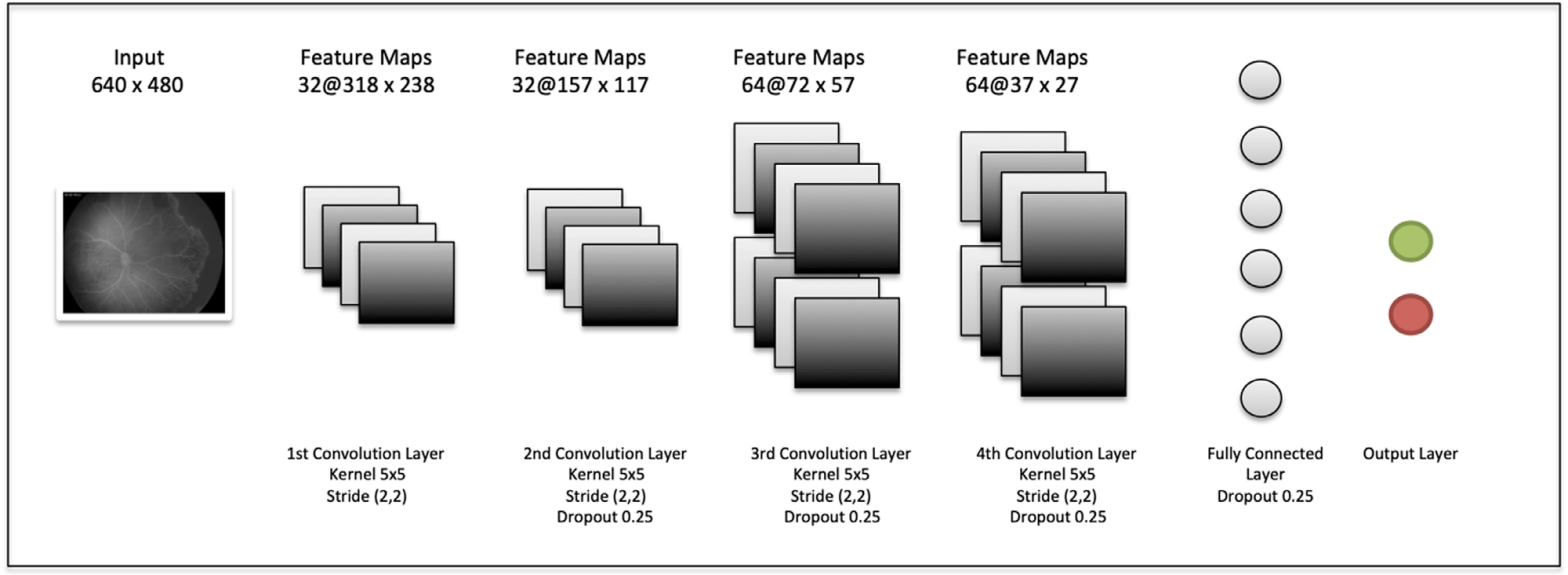
Schematic representation of the convolutional neural network. Different layers from left to right: input layer, four convolutional layers, fully connected layer, and output binary classification layer.

Since 2005, we started to collect FA images of babies undergoing screening for ROP from our institution as well as in other 5 institutions in Italy all parts of the FA team of the Italian ROP Study Group. The image dataset involved a total of 835 de-identified FA images of 149 eyes (90 patients) from 5 centers collected between January 2005 and December 2017. During this time, all infants screened for ROP presenting with retinal vasculature limited in zone I or posterior zone II starting from 31 weeks of postmenstrual age (PMA) underwent FA examination. All images were obtained using Retcam II/III (Natus Medical Incorporated, Pleasanton, CA) with a 130-degree lens following a bolus of 10% fluorescein solution injected intravenously at a dose of 0.1 mL/kg. Only the latest examination sessions performed before 35 weeks of PMA that included at least one image of the posterior pole and one of the periphery with discernible retinal vessels were included in this dataset. In case of treated eyes, we excluded FAs done after treatment. Each eye was associated with a binary outcome: 57 “untreated” eyes and 92 “treated,” with a total of 308 “untreated” and 527 “treated” images. The resolution of the images was 640 and 480 px in 80% of cases (Retcam II), whereas the other 20% had a resolution of 1600 and1200 px (Retcam III). The latter ones were resized to 640 and 480 px before training and no other preprocessing was applied to the entire dataset. A CNN with 4 convolutional layers was trained on a randomly chosen 90% of the images (752 images of 133 eyes). The training and validation sets were split at the patient level to ensure no information leakage from the training. An eye-level classifier was used by the model. Convolutional layers 1 and 2 had 32 filters, whereas convolutional layers 3 and 4 had 64 filters. Every filter in every convolutional layer had a kernel size of 5 × 5 and was applied with a 2 px stride. A dropout layer of 0.25 was applied after every convolutional layer and after the final dense layer. Activation functions were rectified linear units for convolutional layers and dense layer after flattening, and SoftMax Pro Software for the last dense layer. The network was trained for 10 epochs with 25 batch size and the Adam Optimizer (learning rate: 10^−4^, decay: 10^−6^) with categorical cross-entropy as loss function. The accuracy of the prediction was assessed on the remaining 10% of images (83 images of 16 eyes). Details of the network are shown in Figure 1. Keras version 2.2.0 for R with Tensorflow backend version 1.11.0 was used for the analysis. Source code is available at (https://github.com/kbolab/ROP). Additionally, we run the CNN on the same validation set split into “periphery” images and “posterior pole” images. Because zone 1 and posterior zone II were two of the inclusion criteria, it was impossible to isolate the periphery without cropping the images, especially for the zone 1 eyes. Therefore, the “periphery” set included images of the periphery and partial posterior pole, whereas in the “posterior pole” set there were only images of the posterior pole. To get an estimate of the out-of-sample performance of the model, 10-fold cross-validation was performed. The data were partitioned into 10 equally sized folds, and at each iteration the CNN model was trained on 9-folds and tested on the remaining fold on which the receiver operating characteristic (ROC) area under the curve (AUC) value was computed. These 10 values were then averaged to get the mean ROC AUC of the cross-validation and the 95% confidence interval (CIs) were computed as the SD of these 10 values multiplied by the usual 1.96 factor assuming a normal distribution.

## Results

**Figure 2.**
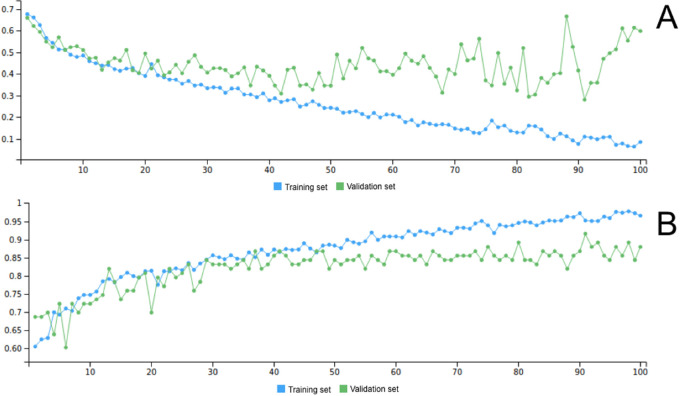
Loss function (**A**) and accuracy (**B**) for training set (*green*) and validation set (*blue*) across 100 epochs.

**Figure 3.**
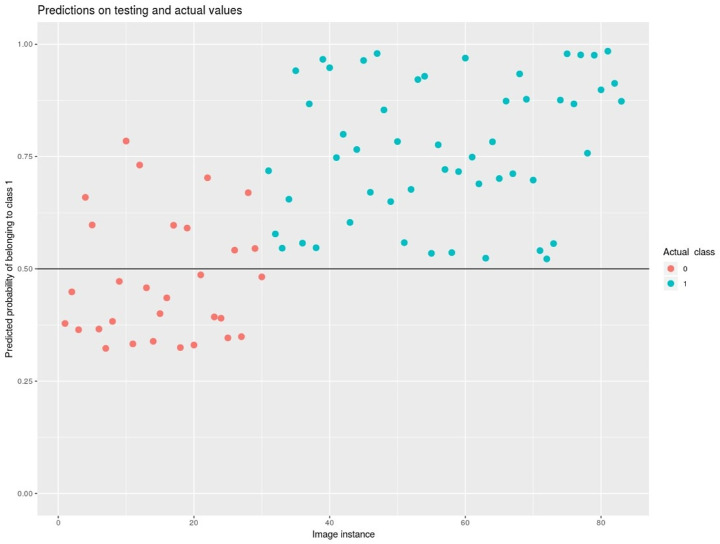
Predicted probability on validation set of belonging to class 1-treated (y-axis). The x-axis represents the single images of the validation set. Colors represent actual class (*blue* for class 1 treated and *red* for class 0 untreated). The graph shows two clusters of data. None of the class 1 treated were predicted as belonging to class 0 untreated, whereas 10 images of 4 eyes of the class 0 untreated were misclassified as class 1 treated.

**Figure 4.**
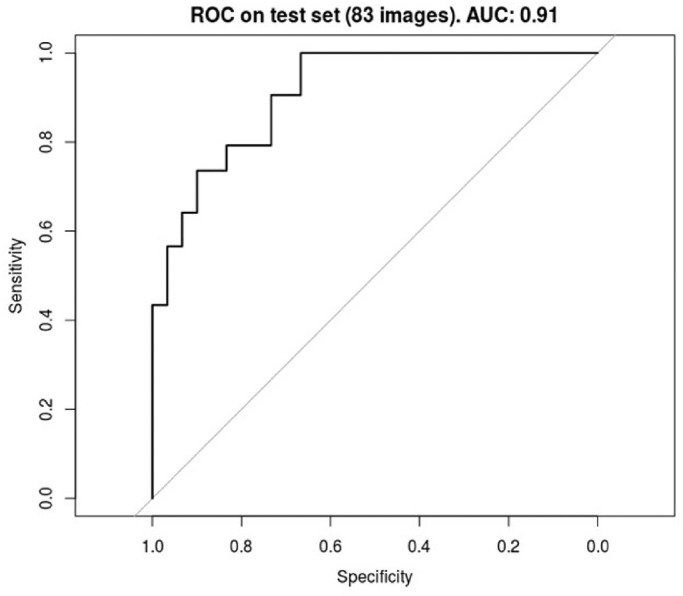
ROC curve and AUC on validation set. ROC, receiver operating characteristic; AUC, area under the curve.

**Figure 5.**
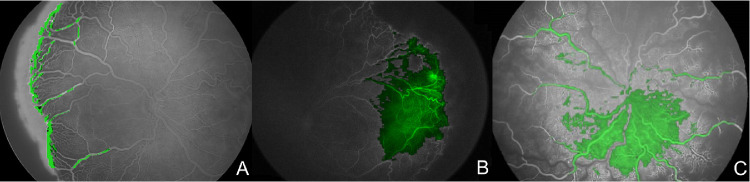
Heatmap of the CNN of three sample images. Activation of the algorithm (*green*) occurs both at the retinal periphery (**A** and **B**) and at the posterior pole (**C**).

**Figure 6.**
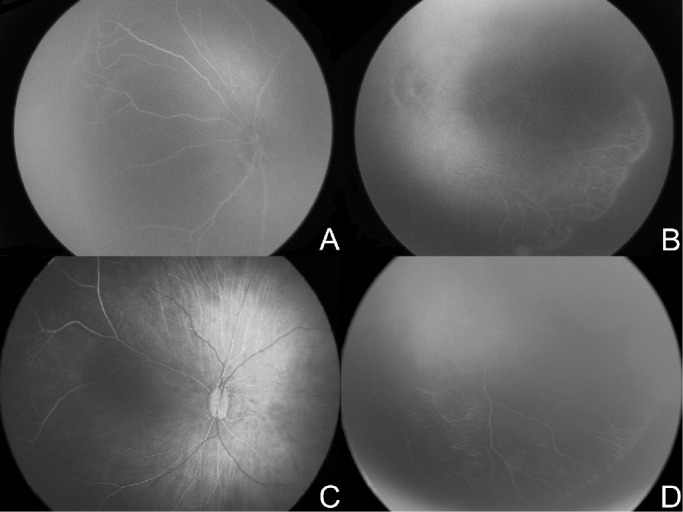
False positive eyes. Angiograms of the four eyes predicted by the algorithm as class 1 treated but actual class 0 untreated.

Among the 149 eyes used to train and assess the CNN, 92 underwent treatment and 57 did not develop treatment requiring ROP. The group that developed treatment requiring ROP were born with a lower gestational age (GA) and body weight (BW; *P* < 0.001) but the PMA at the moment of the FA examination as well as the number of images were comparable (respectively, *P* = 0.249 and *P* = 0.452; Table 1). The number of eyes and corresponding number of images grouped by outcome value (treated / untreated) in training and validation set are reported in Table 2. Graphs of the training and validation set accuracy and loss function for 50 epochs of training are reported in Figure 2. After the model was trained, predictions were made on the validation set images to compute performance metrics other than accuracy. Figure 3 shows the probability that the model assigns to each validation set image of belonging to class 1 (treated): the higher a point is in the graph, the higher the probability of belonging to class 1 according to the model. The blue points, which are the images that actually belong to class 1, generally show a higher probability than the red ones. Indeed, to each blue point is assigned a class-1-probability higher than 50%: a fact that is reflected in the 0.5 thresholded confusion matrix zero false positives rate. In other words, a prediction of “treatment” is always correct on this validation set (Table 3 and Table 4). The AUC of the ROC reached 0.91 (Fig. 4). Our FA-based CNN activates both at the periphery and at the posterior pole in different nodes (Fig. 5). Figure 6 shows the four false positive eyes predicted by the CNN. When we analyzed the dataset separately into “periphery” and “posterior pole” sets, the former yielded a better diagnostic performance with an accuracy of 0.93 compared to 0.75 in the latter one. Ten-fold cross-validation yielded a mean AUC of 0.89 (95% CI = 0.83, 0.95).

**Table 1.** Demographic Data

	Treated (*n* = 92)	Untreated (*n* = 57)	*P Value*
Gestational age, weeks mean ± SD	25.3 ± 1.3	26.8 ± 1.2	<0.001
Birth weight, g mean ± SD	679.2 ± 129.7	833.4 ± 184.3	<0.001
Postmenstrual age at the examination, weeks mean ± SD	32.6 ± 1.2	32.9 ± 1.4	0.249
*N* of images, mean ± SD	5.7 ± 2.5	5.4 ± 2.7	0.452

**Table 2.** Number of Eyes and Corresponding Number of Images Grouped by Outcome Value (Treated/Untreated) in Training and Testing Set

Training Set	*N* Eyes	*N* Images
Untreated (0)	51	278
Treated (1)	82	474
Total	133	752
**Testing set**	*N* Eyes	*N* Images
Untreated (0)	6	30
Treated (1)	10	53
Total	16	83

**Table 3.** Confusion Matrix on Testing Set at the Eye Level and Image Level

		True Negatives	True Positives
Eye level	Predicted negatives	4	0
	Predicted positives	2	10
Image level	Predicted negatives	20	0
	Predicted positives	10	53

**Table 4.** Confusion Matrix Statistics on Testing Set at the Eye Level and Image Level

	Accuracy	Kappa	Sensitivity	Specificity	PPV	NPV
Eye level	0.88	0.71	1.00	0.67	0.83	1.00
Image level	0.88	0.72	1.00	0.67	0.84	1.00

PPV, positive predictive value; NPV, negative predictive value.

## Discussion

The early treatment for the ROP study set Plus disease as the predominant factor in the indication for treatment, diverting the focus to the posterior pole of the retina. Over the years, massive effort has been put to standardize Plus disease by many groups around the world. The work done by the Imaging and Informatics in Retinopathy of Prematurity (i-ROP) Research Consortium has been particularly valuable.^4^^–^^6^ Apart from the large number of retinal images collected, their studies were mainly focused on Plus disease: inter alia they introduced the concept of Plus disease as a continuum spectrum of progression from normal to unequivocally pathologic.^7^ This was the premise to the introduction of deep learning (DL) into the ROP management. The results of the application of this technology are absolutely encouraging.^4^^,^^8^ In contrast to iROP, our study tries to shift the attention to the whole retina, in contrast to the posterior pole alone, just having in mind that ROP is a vascular disease that occurs at the edge of the vascularized retina, thus in the periphery. When we tested our CNN on the split datasets we yielded a higher diagnostic performance with the “periphery” set. This reflects the additional (not substitutive) value of the periphery that should not be overlooked. To show the modification of peripheral retinal circulation, color fundus photographs are often insufficient to capture details of the pathologic process, whereas FA seems to be more efficient for this purpose. FA in the evaluation of ROP was introduced in the 1970s by Flynn and Kushner^9^^,^^10^ and Cantolino et al.,^11^ recognizing that ROP is a mainly vascular disease from the start. There are some caveats about the use of FA in the management of ROP, especially for the indication to treat. There is no exact correspondence in ROP diagnosis defined by the International Classification of ROP (ICROP)^12^ and FA findings. Furthermore, all the major randomized controlled trials that defined the guidelines for treatment did not include FA.^13^ Although no adverse event related to the procedure has been reported for years at our centers, FA has to be considered invasive for such delicate babies. For the same reason, large scale validation per se will be difficult.

In 2015, Klufas et al. showed an improvement of the inter-expert agreement on ROP diagnosis using FA, probably related to better visualization of vessel shape and contour as well as amount of peripheral dye leakage.^14^ Recently, Mansukhani et al. described the differences on FA between eyes treated with intravitreal injection of bevacizumab and eyes that regressed without treatment and interestingly found frequent abnormal vascular patterns in both groups.^15^ This and many other reports showed that the use of FA might play a paramount role in the long-term follow-up management of babies treated with antivascular endothelial growth factor (VEGF) agents.^15^^–^^18^

Furthermore, it is putative that information obtained using FA may provide greater understanding of subtleties of acute-phase ROP.^14^^,^^19^^,^^20^

We hypothesized that FA images could be a potential dataset to train a DL algorithm to detect ROP cases requiring treatment. We applied DL to nonprocessed whole retina FA images in order to demonstrate the feasibility of this analysis to this imaging technique for the first time. With our study, we demonstrated that even a minimal number of FA images of the premature babies’ retinas can train a CNN with good ROC as well as accuracy. Because 20% of the FA images had a resolution of 1600 and 1200 px, whereas the remaining 80% had a resolution of 640 and 480 px, we decided to design and build the network with the input layer dimension equal to 640 and 480. Thus, we resized the high-resolution images before they were fed into the network. This allowed to keep the vast majority of images to their original resolution, and at the same time to reduce the number of parameters compared to a network with a bigger size of input, and consequently to reduce the training time and processing unit requirements. Moreover, we decided to train the network from scratch on an architecture that we customized from the basic blocks of the CNNs settings. We tested whether a simple architecture CNN with no transfer-learning involved could still reach acceptable or good performance for this kind of classification task. As to the performances, we have to stress that one of the problems with machine learning models, including DL, is overfitting. This can occur when the trained model does not generalize well to unseen cases, but fits the training data well, especially when the training sample size is small. The CNN in this study used dropout regularization strategies to help overcome this issue. Moreover, the shape of the training curve (see Fig. 2) can be used to assess the occurrence of overfitting. From the curve, we can see that the loss function is similar in trend and values for both validation and training datasets, which indicates well-fit curves. The term “prediction” in the field of machine learning does not always refer to forecasting a future outcome. Rather, it alludes to the output of a model after it has been trained on a dataset and applied to a different dataset. We included in our study both FAs done before the development of ROP requiring treatment and those performed when eyes were deemed treatment-requiring, right before intervention. To develop an algorithm that was able to predict, in the common sense of the word, a future outcome, we would need to include the former category only. We were not able to do that because of the limited sample size. Future work will compare these results in terms of performances and methodology with another state-of-the-art network architecture, as well as validation of the model output on new FA images. Further studies are also needed to exploit different fields of applications, such as analysis of retinal vasculature images from humans and even from animal models of ROP with other imaging devices.

FA team of the Italian ROP study Group:

Writing committee: Domenico Lepore, MD, Marco H. Ji, MD, Monica M. Pagliara, MD, Jacopo Lenkowicz, Nikola D. Capocchiano, MD, Luca Tagliaferri, MD, Luca Boldrini, MD, Vincenzo Valentini, MD, and Andrea Damiani, PhD.

Participating Clinical Centers:

Catholic University of Sacred Heart, Fondazione Policlinico Agostino Gemelli IRCSS (Rome, Italy). Principal Investigator Domenico Lepore, MD; Marco H. Ji, MD; Monica M. Pagliara, MD; Fernando Molle, MD; Antonio Baldascino, MD; Velia Porcaro, MD; Patrizia Papacci, MD; and Giovanni Vento, MD.

University of Verona (Verona, Italy). Principal Investigator Elena Gusson, MD, and Giorgio Marchini, MD.

University of Udine (Udine, Italy). Principal Investigator Silvia Pignatto, MD, and Paolo Lanzetta, MD.

ASST Great Metropolitan Niguarda Hospital (Milan, Italy). Principal Investigator Elena Piozzi, MD, Marco Mazza, MD, and Giovanni Marsico, MD.

Meyer Children's Hospital (Florence, Italy). Principal Investigator Giuseppina Fortunato, MD, and Roberto Caputo, MD.
